# Stress eating: an online survey of eating behaviours, comfort foods, and healthy food substitutes in German adults

**DOI:** 10.1186/s12889-022-12787-9

**Published:** 2022-02-24

**Authors:** Kathrin Gemesi, Sophie Laura Holzmann, Birgit Kaiser, Monika Wintergerst, Martin Lurz, Georg Groh, Markus Böhm, Helmut Krcmar, Kurt Gedrich, Hans Hauner, Christina Holzapfel

**Affiliations:** 1 Institute for Nutritional Medicine, School of Medicine, Technical University of Munich, Georg-Brauchle-Ring 62, 80992 Munich, Germany; 2 Research Group Public Health Nutrition, ZIEL – Institute for Food & Health, Technical University of Munich, Freising, Germany; 3 Research Group Social Computing, Department of Informatics, Technical University of Munich, Garching, Germany; 4 Krcmar Lab, Department of Informatics, Technical University of Munich, Garching, Germany; 5 Department of Informatics, University of Applied Sciences Landshut, Landshut, Germany; 6 Else Kröner Fresenius Center for Nutritional Medicine, School of Life Sciences, Technical University of Munich, Freising, Germany

**Keywords:** Stress, Nutrition, Chocolate, Coffee, Obesity

## Abstract

**Background:**

In many people, stress is associated with changes in eating behaviour. Food products consumed during stress (comfort foods) are often unhealthy. It is rather unknown what comfort foods are consumed in Germany and what healthier food products are considered as alternatives to support stress-eaters in making healthier food choices.

**Methods:**

This online survey was conducted in spring 2021 throughout Germany. Participants were digitally recruited by newsletters, homepages, social media, and mailing lists. The survey included a standardized questionnaire with items concerning e.g. sociodemography, stress, and nutrition. Comfort foods were pre-selected through literature search and food substitutes were defined and discussed by experts. Analyses examined comfort food consumption and substitute preferences dependent on sex, age, body mass index (BMI), and being a self-identified stress-eater. The statistical analysis was performed using R.

**Results:**

Survey participants were mostly female (80.6%, 994/1234), had a mean age of 31.4 ± 12.8 years and a mean BMI of 23.4 ± 4.3 kg/m^2^. Participants stated, that the two favourite comfort foods were chocolate (consumed *often/very often* by 48.3%, 596/1234) and coffee (consumed *often/very often* by 45.9%, 566/1234). Regarding food substitutes, the most frequently named alternative food for chocolate and cookies was fresh fruits (for chocolate: 74.4%, 815/1096, for cookies: 62.6%, 565/902). Tea without added sugar (64.4%, 541/840) was the preferred substitute for coffee. Almost 50% of participants (48.1%, 594/1234) identified themselves as stress-eaters, of which 68.9% (408/592) stated to eat (very) often more than usual in subjective stress situations.

**Conclusions:**

The results from this work suggest that specific comfort foods and substitutes are preferred by the participants in stressful situations. This knowledge about food choices and substitutes should be investigated in further studies to improve eating behaviour in stressful situations.

**Trial registration:**

The survey was registered in the German Register of Clinical Studies (Registration number: DRKS00023984).

**Supplementary Information:**

The online version contains supplementary material available at 10.1186/s12889-022-12787-9.

## Introduction

Stress is known to be associated with disturbed sleeping, memory, learning, and attention and can have a negative impact on the immune and the cardiovascular system [1]. It is also known that stress can have an indirect effect on health, e.g. by changing eating behaviour [2]. For stress recognition, different stress indicators and assessment methods can be used [3].

Previous studies have shown that nearly equal numbers of participants (i.e. 50%) responded to stress by eating either more or less [4–6]. Moreover, stress seems to change food preferences towards unhealthy food products. A systematic review of 16 studies examining food intake and food frequency of women under psychological stress has shown a significant association between stress and an unhealthy diet (e.g. high in fat, sweets, and salt but low in fruits and vegetables) [7]. In addition, a meta-analysis of five studies with 3471 participants revealed a negative association between stress and diet quality [7].

Certain mediators seem to have an impact on the relationship between stress and eating. Eating self-regulation was shown to partially influence the relationship of stress and emotional eating [8]. Van Blyderveen et al. could show in female undergraduated students that impulsive women had a higher susceptibility to stress-induced eating, and that impulsivity and emotional suppression had an influence on the relationship between negative affect and food consumption during stress [9]. A review by Adam et al. describes the mediator role of the reward system in the relationship between stress and stress-induced eating [10]. Severe stress can result in higher cortisol levels, leading to activation of the hypothalamus pituitary adrenal axis, which interacts with various hormones having an influence on food intake [10].

Experiencing stress accompanied by negative emotions is one reason why so called “comfort foods” are consumed [11]. Comfort foods are suspected to be comforting by having positive emotional effects. An experiment by Wagner et al. showed that consuming your personal comfort food has a positive emotional effect, but not more than other food or no food [12]. A review summarised that there is not enough evidence for the reasons of consuming comfort foods and their emotional benefits [13].

The selection of comfort foods is dependent on country-specific popular foods, why comparisons across countries are limited. In studies from Korea [14], Saudi Arabia [15], Great Britain [5], the USA [16, 17], and in a study comparing three different European countries including Germany (*N* = 696) [18] a variation of comfort foods has been described. In addition an association between perceived stress and frequent consumption of unhealthy food products like sweets and fast food could be shown in these studies. Moreover, it is suggested that comfort food preferences are associated with gender [5, 15, 18].

The study examining comfort foods in Germany [18] was focused on students and used a non-validated food frequency questionnaire to measure food consumption.

A frequent consumption of energy-dense comfort foods during stress could lead to an increase of caloric intake and, as a consequence of a chronically positive energy balance, to increased body weight. The MIDUS study in US adults observed an association between stress-eating and metabolic parameters, which could be attributed to the presence of abdominal obesity [19]. Increased body weight in turn could lead to weight stigmatization, which could result in increased food intake forming a vicious circle [20]. It should be investigated whether a positive association between BMI and comfort food consumption frequency exists.

In conclusion, the recommendation of healthier alternatives for comfort foods (substitutes) could be a prerequisite to prevent from unhealthy energy-dense food intake in stressful situations. To put this into practice, concepts of nudging are able to positively influence peoples’ behaviour [21]. To the best of our knowledge there are no studies that investigated whether stress-eaters would consume substitutes instead of comfort foods if offered to them under stress.

Aim of the present online survey was to collect sex-, age-, BMI-, and stress-eater-specific data on food products, which are chosen in stressful situations, among adults in Germany together with information on food products which are considered as healthier alternatives.

## Methods

### Survey

Data were collected by an open online survey performed during the Covid-19 pandemic between January and April 2021 throughout Germany. Participants were digitally recruited by university internal and external channels such as newsletters, homepages, social media accounts (e.g. facebook), and mailing lists using snowball sampling. Interested persons were guided to the online survey by an invitation including a link to the survey platform SoSci Survey (V3.1.06).

A calculation of the response rate was not possible since the survey invitation was delivered electronically and the number of invitations was unknown.

The survey started with an introduction presenting information about the research team, aim of the survey, guidance on answering the questions, and information about data privacy and protection. Before answering the survey questions, participants had to confirm the data privacy statement and to give informed consent prior to participation. Additional inclusion criteria were being 18+ years old and having good German language skills. No incentives were offered to the participants.

### Questionnaire

The 38-item questionnaire was developed by an interdisciplinary team of nutritionists, public health experts, and computer scientists. The questionnaire was implemented in SoSci Survey and pretested by the target group with regard to understanding, difficulty, and structure of questions and answers. According to the results from the pretest the questionnaire was shortend and single questions were changed. The final questionnaire comprised questions (closed, open, single or multiple choice) referring to nutrition (one question), stress perception and coping (four questions), stress-eating (17 questions), technical behaviour (four questions), digital applications (apps) detecting stress (three questions), and personality (one question) from which a selection of questions focused on stress-eating behaviour is presented in this work. Socio-demographic and anthropometric data (eight questions) were collected at the end of the survey. Neutral answer options like “sometimes” and “other” were provided if indicated. Each question had to be answered to continue, whereas the survey could be stopped after each question.

### Comfort foods

**Fig. 1:**
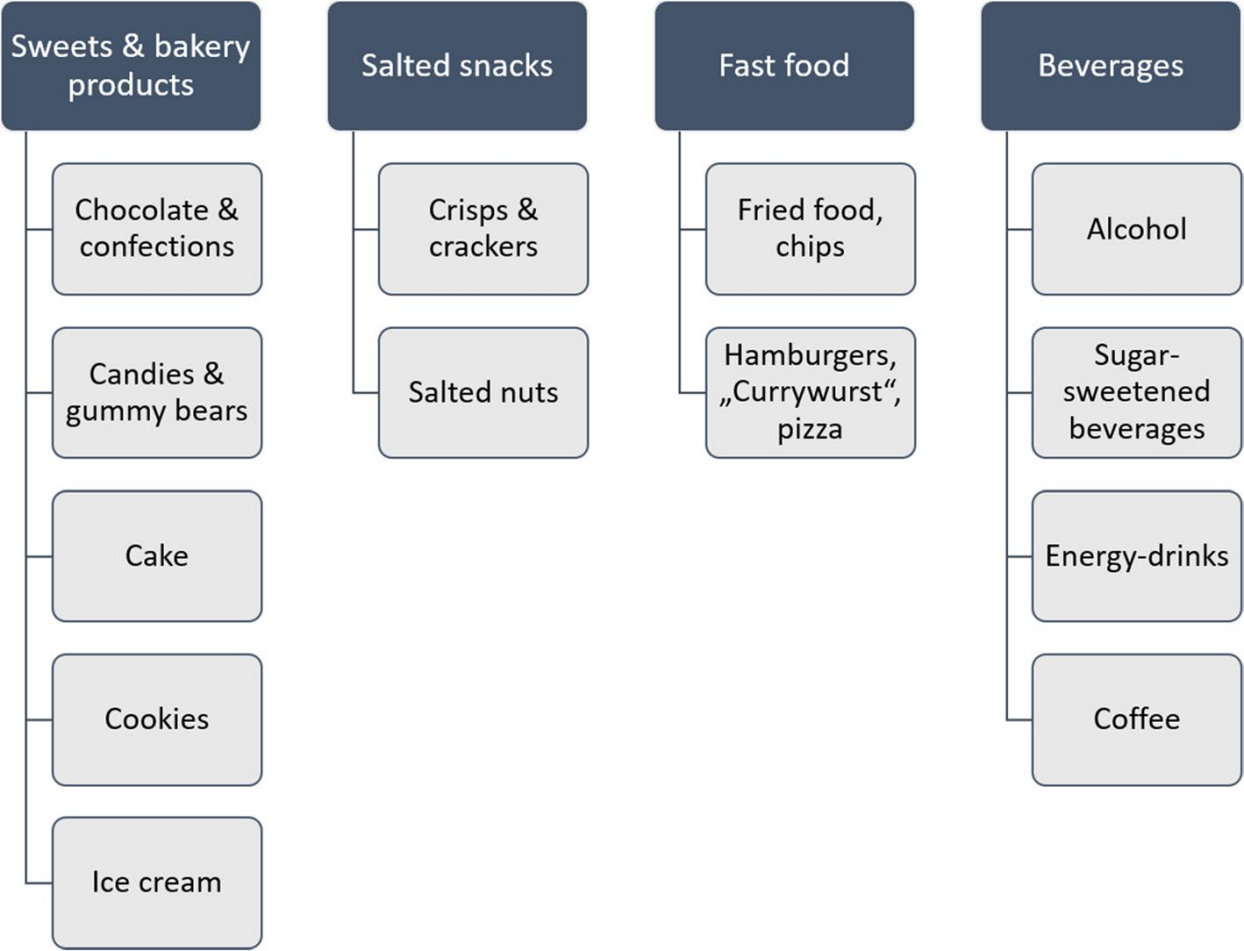
Pre-selected comfort foods identified by literature search and adapted to German food culture

Literature search was performed to pre-select comfort foods and to divide them in four food product categories. Studies about stress-eating and comfort foods were selected [5, 14–18, 22–25]. Most of the studies were conducted in the USA and most of the participants were students. The selected studies had no uniform definition of a comfort food. Therefore, the final set of 13 comfort foods was adapted to German food culture according to experts’ opinion. Figure 1 shows the pre-selected comfort foods, which have been integrated into the questionnaire. In the survey, participants were asked how frequently (5-Likert scale: “never” to “very often”) they consume these comfort foods in stressful situations. Comfort foods other than pre-selected could not be specified by survey participants. This question had been answered by all participants, not only by those who identified themselves as stress-eaters.

### Substitutes

Suitable substitutes for each comfort food were discussed and pre-selected by the same experts who pre-selected the comfort foods. The following criteria were considered: substitutes should 1) not be comfort foods, 2) have a similar flavour like the comfort foods, and 3) be “healthier” (e.g. less energy, salt, or sugar) than the comfort foods. Quantity, volume, brand, and packaging of substitutes were not considered. In addition to the pre-selected substitutes, survey participants could name other substitutes. Participants were asked whether they could imagine to consume the offered substitutes for the prevailing comfort foods they stated to consume in stressful situations.

### Stress-eating

Participants were asked if they would identify themselves as stress-eaters (*“Do you think you are a stress-eater?”*). Being a stress-eater was defined by eating differently when experiencing stress. Survey participants who stated to be a stress-eater where asked to characterise their stress-eating behaviour (e.g. eating more, less, more often, etc.). Another analysis of this survey by Kaiser et al. used the validated *Salzburg Stress Eating Scale* to characterize stress-overeaters [26].

### Statistical analysis

As a first step, integrity and plausibility checks were performed. Descriptive data analyses (frequencies, percentages, standard deviation, and mean) were performed using Excel 2016 (Microsoft Corp). Only data from participants who provided sociodemographic data were analysed. Since the dataset contains missing answers and some single answers had to be excluded because of inconsistencies in the answers, sample size differs between questions and answer options. Normality was tested using the Shapiro-Wilk test. Variance homogeneity was checked by using F-test. Differences in mean age and BMI were estimated by performing Welch’s t-test or a two-sample t-test. Pearson’s Chi-squared test or Fisher’s exact test were used to examine sex differences in stress-eating behaviour and differences between self-identified stress-eaters and non-stress-eaters. Associations between sex, age, BMI, and being a self-identified stress-eater and the consumption of comfort foods and the consideration of named substitutes were assessed for total of participants using multinomial logistic regression analysis or binary logistic regression analysis. In addition, a sub-analysis in self-identified stress-eaters was performed. After adjusting for multiple testing by Bonferroni correction, *p*-values < 0.004 for multinomial and <  0.002 for binary logistic regression models were considered as statistically significant. All analyses were performed using R (V4.1.0).

## Results

### Characteristics

Characteristics of the survey population are presented in Table 1. Participants were on average 31.4 ± 12.8 years old and had a mean BMI of 23.4 ± 4.3 kg/m^2^. Women showed a significantly lower BMI compared to men (23.10 vs. 24.60 kg/m^2^, *p* = 1.07e^− 6^). Most participants were female (80.6%), single (66.0%), students (53.3%), and had a higher education (82.0%). Regarding stress frequency, 22.9% and 41.8% of participants reported being stressed everyday or more than once a week, respectively. The two most frequently indicated stressors were work (65.2%) and partner, family, and friends (36.1%). Half of the survey participants (48.1%) identified themselves as stress-eaters. Self-identified stress-eaters were predominantly women (88.7%, 527/594) (Table 1).

**Table 1:** Characteristics of the study population

	Total (*N* = 1234)	Females (*N* = 994)	Males (*N* = 240)	Stress-eaters (*N* = 594)
	n (%) or mean (± SD)	n (%) or mean (± SD)	n (%) or mean (± SD)	n (%) or mean (± SD)
Age in years^a^	31.4 (± 12.8)	31.3 (± 12.5)	32.1 (± 14.2)	31.1 (± 12.3)
BMI in kg/m^2bc^	23.4 (± 4.3)	23.1 (± 4.3)	24.6 (± 4.1)	24.4 (± 4.8)
Underweight (< 18.5)	65 (5.3)	60 (6.0)	5 (2.1)	12 (2.0)
Normal weight (18.5 – 24.9)	839 (68.0)	704 (70.8)	135 (56.3)	379 (63.8)
Overweight (25.0 – 29.9)	234 (19.0)	160 (16.1)	74 (30.8)	132 (22.2)
Obesity (≥ 30.0)	96 (7.9)	70 (7.0)	26 (10.8)	71 (12.0)
Marital status^d^				
Married	284 (23.0)	233 (23.4)	51 (21.3)	138 (23.2)
Single	815 (66.0)	642 (64.6)	173 (72.1)	393 (66.2)
Other	135 (10.9)	119 (12.0)	16 (6.7)	63 (10.6)
Highest general school certificate^e^				
General/subject-related higher education entrance qualification^f^	1012 (82.0)	821 (82.6)	191 (79.6)	487 (82.0)
Other	222 (18.0)	173 (17.4)	49 (20.4)	107 (18.0)
Profession^g^				
Employee/official	431 (34.9)	368 (37.0)	63 (26.3)	212 (35.7)
Student	658 (53.3)	509 (51.2)	149 (62.1)	318 (53.5)
Other	145 (11.8)	117 (11.8)	28 (11.7)	64 (10.8)

^a^How old are you?

^b^How tall are you?

^c^What is your weight?

^d^What is your marital status?

^e^What is your highest general school certificate?

^f^Including German Abitur (diploma from German secondary schools qualifying for university admission)

^g^What is your predominant working at the moment?

The stress-eating behaviour of self-identified stress-eaters is shown in Table 2. More than 50% of the participants stated to eat more (*often/very often*: 68.9%, 408/592), more often (*often/very often*: 67.2%, 399/594), faster (*often/very often*: 55.6%, 330/594), or other foods (*often/very often*: 54.4%, 323/594) in stressful situations. There were no statistically significant sex differences (Table 2, Additional File 1: Supplementary Table 1).

**Table 2:** Stress-eating behaviour of participants who identified themselves as stress-eaters (*N* = 594)^a^

	Never	Rarely	Sometimes	Often	Very often	*p*-value
“I eat …”	n (%)	n (%)	n (%)	n (%)	n (%)	
More*	16 (2.7)	32 (5.4)	136 (23.0)	270 (45.6)	138 (23.3)	0.08
Less*	197 (33.4)	216 (36.6)	103 (17.5)	51 (8.6)	23 (3.9)	0.82
More often	31 (5.2)	47 (7.9)	117 (19.7)	280 (47.1)	119 (20.0)	0.12
More rarely	233 (39.2)	205 (34.5)	85 (14.3)	53 (8.9)	18 (3.0)	0.88
Faster	63 (10.6)	79 (13.3)	122 (20.5)	192 (32.3)	138 (23.2)	0.38
Slower	341 (57.4)	166 (27.9)	62 (10.4)	19 (3.2)	6 (1.0)	0.89
Other food	50 (8.4)	67 (11.3)	154 (25.9)	189 (31.8)	134 (22.6)	0.55

*Different sample sizes because of missing answers

^a^What applies to you regarding stress-eating?

### Comfort foods

The consumption frequency of 13 pre-selected comfort foods in stressful situations is shown in Table 3. Regarding all participants, the two most often consumed comfort foods were chocolate (*often/very often*: 48.3%) and coffee (*often/very often*: 45.9%). The comfort foods, which were stated to be never consumed in stressful situations by at least 50% of the survey participants were energy drinks (88.9%), fried food (62.1%), ice cream (59.3%), sugar-sweetened beverages (58.1%), hamburgers (52.8%), and candies (50.8%). Supplementary Table 2 (Additional File 1) shows the sex-specific consumption frequency of comfort foods.

**Table 3:** Consumption frequency of pre-selected comfort foods (*N* = 1234)^a^

	Never	Rarely	Sometimes	Often	Very often
	n (%)	n (%)	n (%)	n (%)	n (%)
Chocolate	138 (11.2)	221 (17.9)	279 (22.6)	356 (28.8)	240 (19.4)
Candies	627 (50.8)	283 (22.9)	167 (13.5)	108 (8.8)	49 (4.0)
Ice cream	732 (59.3)	264 (21.4)	159 (12.9)	59 (4.8)	20 (1.6)
Cake	413 (33.5)	332 (26.9)	306 (24.8)	147 (11.9)	36 (2.9)
Cookies	332 (26.9)	276 (22.4)	328 (26.6)	231 (18.7)	67 (5.4)
Crisps/crackers	474 (38.4)	303 (24.6)	224 (18.2)	172 (13.9)	61 (4.9)
Salted nuts	614 (49.8)	253 (20.5)	199 (16.1)	123 (10.0)	45 (3.6)
Fried food/chips	766 (62.1)	236 (19.1)	134 (10.9)	76 (6.2)	22 (1.8)
Hamburgers etc.	651 (52.8)	270 (21.9)	184 (14.9)	104 (8.4)	25 (2.0)
Alcohol*	590 (47.9)	268 (21.8)	229 (18.6)	107 (8.7)	37 (3.0)
Sugary beverages	717 (58.1)	234 (19.0)	156 (12.6)	77 (6.2)	50 (4.1)
Energy drinks	1097 (88.9)	60 (4.9)	31 (2.5)	22 (1.8)	24 (1.9)
Coffee	394 (31.9)	104 (8.4)	170 (13.8)	279 (22.6)	287 (23.3)

*Different sample size because of missing answers.* N *= 1231

^a^Think about the last month. How often did you eat the following food when you experienced stress?

In a sub-analysis of self-identified stress-eaters, chocolate (*often/very often*: 72.4%, 430/594) and coffee (*often/very often*: 53.3%, 318/594) were again found as the two most often consumed comfort foods (Supplementary Table 2). Regarding the consumption frequency of these comfort foods, a statistically significant difference was found between self-identified stress-eaters and non-stress-eaters (*p* <  0.001).

The odds of stating to eat chocolate was three times higher for women (*very often*: OR = 3.05, *p* <  0.001) than for men. Regarding age and BMI statistically significant differences have been observed (Table 4). For each comfort food except from energy drinks and sugar-sweetend beverages, it could be shown that stress-eaters stated up to 25 times more often to consume the comfort food (very) frequently during stress compared to non-stress-eaters (*p* <  0.001) (Table 4).

**Table 4:** Consumption frequency of comfort foods according to sex, age, BMI, and being a stress-eater

		Sex^a^		Age		BMI		Stress-eaters^f^	
Comfort food	Frequency	OR [95% CI]^b^	p^c^	OR [95% CI]	p^d^	OR [95% CI]	p^e^	OR [95% CI]	p^g^
Chocolate	never	–	–	–	–	–	–	–	–
	rarely	1.22 [0.75; 1.97]	0.40	1.01 [0.99; 1.03]	0.29	1.02 [0.95; 1.09]	0.57	2.19 [1.20; 3.99]	0.01
	sometimes	2.78 [1.69; 4.57]	< 0.001	1.06 [0.99; 1.03]	0.11	1.06 [0.99; 1.13]	0.09	2.50 [1.41; 4.45]	0.002
	often	2.07 [1.26; 3.41]	0.0041	1.05 [0.99; 1.03]	0.28	1.05 [0.98; 1.12]	0.15	10.60 [6.06; 18.53]	< 0.001
	very often	3.05 [1.66; 5.59]	< 0.001	1.07 [0.98; 1.02]	0.95	1.07 [1.00; 1.15]	0.046	23.52 [12.76; 43.38]	< 0.001
Candies	never	–	–	–	–	–	–	–	–
	rarely	1.25 [0.85; 1.81]	0.25	1.00 [0.99; 1.01]	0.76	1.04 [1.00; 1.08]	0.03	1.56 [1.15; 2.10]	0.0038
	sometimes	1.25 [0.79; 1.97]	0.34	1.01 [0.99; 1.03]	0.08	1.07 [1.03; 1.12]	0.002	1.50 [1.04; 2.17]	0.03
	often	1.10 [0.61; 2.00]	0.75	1.03 [1.01; 1.04]	< 0.001	1.06 [1.01; 1.12]	0.02	6.43 [3.79; 10.93]	< 0.001
	very often	1.43 [0.57; 3.57]	0.44	0.99 [0.97; 1.02]	0.70	1.10 [1.03; 1.17]	0.0043	7.99 [3.45; 18.51]	< 0.001
Ice cream	never	–	–	–	–	–	–	–	–
	rarely	0.96 [0.67; 1.39]	0.84	1.00 [0.99; 1.01]	0.77	1.04 [0.99; 1.07]	0.06	1.84 [1.36; 2.49]	< 0.001
	sometimes	1.22 [0.74; 2.00]	0.43	0.99 [0.98; 1.01]	0.34	1.05 [1.00; 1.09]	0.03	3.34 [2.27; 4.92]	< 0.001
	often	2.09 [0.80; 5.46]	0.13	0.99 [0.97; 1.01]	0.41	1.02 [0.96; 1.09]	0.52	4.56 [2.41; 8.66]	< 0.001
	very often	4.31 [0.56; 33.26]	0.16	0.97 [0.92; 1.01]	0.17	1.09 [1.00; 1.19]	0.05	3.49 [1.22; 10.02]	0.02
Cake	never	–	–	–	–	–	–	–	–
	rarely	1.76 [1.20; 2.58]	0.0038	1.02 [1.00; 1.03]	0.009	1.01 [0.97; 1.05]	0.67	1.86 [1.35; 2.55]	< 0.001
	sometimes	1.38 [0.94; 2.03]	0.10	1.03 [1.01; 1.04]	< 0.001	0.99 [0.95; 1.03]	0.71	2.49 [1.80; 3.46]	< 0.001
	often	2.20 [1.22; 3.99]	0.01	1.03 [1.01; 1.04]	0.009	1.02 [0.97; 1.07]	0.52	5.36 [3.45; 8.34]	< 0.001
	very often	2.04 [0.68; 6.13]	0.20	1.03 [1.01; 1.06]	0.02	1.04 [0.97; 1.12]	0.24	11.65 [4.30; 31.61]	< 0.001
Cookies	never	–	–	–	–	–	–	–	–
	rarely	1.21 [0.81; 1.80]	0.35	1.02 [1.00; 1.03]	0.01	1.00 [0.95; 1.04]	0.92	1.78 [1.24; 2.56]	0.002
	sometimes	1.58 [1.05; 2.37]	0.03	1.01 [0.99; 1.03]	0.08	1.00 [0.96; 1.05]	0.86	2.83 [2.01; 4.00]	< 0.001
	often	1.16 [0.73; 1.83]	0.54	1.00 [0.99; 1.02]	0.55	1.01 [0.97; 1.06]	0.64	7.20 [4.83; 10.72]	< 0.001
	very often	2.31 [0.86; 6.20]	0.10	0.99 [0.96; 1.01]	0.38	1.01 [0.94; 1.08]	0.85	25.31 [10.38; 61.69]	< 0.001
Crisps	never	–	–	–	–	–	–	–	–
	rarely	1.24 [0.84; 1.82]	0.27	0.99 [0.98; 1.01]	0.27	1.05 [1.01; 1.10]	0.009	1.34 [0.98; 1.82]	0.07
	sometimes	1.08 [0.71; 1.63]	0.73	0.98 [0.97; 0.99]	0.01	1.07 [1.03; 1.12]	0.002	1.52 [1.08; 2.15]	0.02
	often	0.80 [0.49; 1.29]	0.36	0.99 [0.97; 1.00]	0.17	1.05 [1.00; 1.10]	0.05	5.46 [3.60; 8.30]	< 0.001
	very often	0.89 [0.42; 1.91]	0.77	0.97 [0.94; 0.99]	0.02	1.09 [1.02; 1.18]	0.008	8.22 [3.95; 17.12]	< 0.001
Salted nuts	never	–	–	–	–	–	–	–	–
	rarely	0.77 [0.53; 1.12]	0.17	1.01 [0.99; 1.02]	0.11	1.03 [0.99; 1.07]	0.11	1.40 [1.02; 1.92]	0.04
	sometimes	1.35 [0.85; 2.17]	0.21	1.01 [0.99; 1.03]	0.08	1.01 [0.97; 1.05]	0.67	1.65 [1.17; 2.32]	0.004
	often	0.81 [0.48; 1.36]	0.42	1.03 [1.01; 1.04]	< 0.001	1.01 [0.96; 1.06]	0.82	2.34 [1.53; 3.58]	< 0.001
	very often	0.38 [0.18; 0.79]	0.009	1.02 [0.99; 1.04]	0.12	1.06 [0.99; 1.13]	0.06	8.81 [3.70; 20.98]	< 0.001
Fried food	never	–	–	–	–	–	–	–	–
	rarely	0.96 [0.65; 1.42]	0.82	1.00 [0.99; 1.01]	0.77	1.03 [0.99; 1.07]	0.08	1.45 [1.06; 1.98]	0.02
	sometimes	0.70 [0.44; 1.11]	0.13	0.99 [0.97; 1.00]	0.09	1.07 [1.02; 1.11]	0.005	1.56 [1.05; 2.32]	0.03
	often	0.79 [0.41; 1.50]	0.46	0.98 [0.96; 0.99]	0.05	1.09 [1.04; 1.15]	< 0.001	3.90 [2.19; 6.94]	< 0.001
	very often	0.35 [0.13; 0.94]	0.04	0.91 [0.84; 0.98]	0.008	0.99 [1.05; 1.21]	0.06	5.13 [1.74; 15.11]	0.003
Hamburgers	never	–	–	–	–	–	–	–	–
	rarely	0.85 [0.58; 1.24]	0.40	0.99 [0.99; 1.01]	0.80	1.05 [1.01; 1.09]	0.01	1.01 [0.75; 1.37]	0.94
	sometimes	0.53 [0.35; 0.80]	0.003	0.97 [0.95; 0.98]	< 0.001	1.09 [1.04; 1.13]	< 0.001	2.03 [1.42; 2.92]	< 0.001
	often	0.55 [0.32; 0.95]	0.03	0.96 [0.94; 0.98]	< 0.001	1.11 [1.05; 1.16]	< 0.001	2.93 [1.82; 4.72]	< 0.001
	very often	0.67 [0.21; 2.11]	0.49	0.94 [0.89; 0.99]	0.02	1.08 [0.98; 1.19]	0.12	7.01 [2.27; 21.63]	< 0.001
Alcohol	never	–	–	–	–	–	–	–	–
	rarely	1.15 [0.78; 1.69]	0.48	1.01 [0.99; 1.02]	0.26	1.02 [0.99; 1.06]	0.21	1.05 [0.77; 1.42]	0.78
	sometimes	1.28 [0.82; 1.93]	0.29	1.02 [1.01; 1.04]	< 0.001	0.99 [0.96; 1.04]	0.88	1.37 [0.99; 1.90]	0.06
	often	0.71 [0.43; 1.18]	0.18	1.01 [0.99; 1.03]	0.15	1.06 [1.01; 1.11]	0.01	1.70 [1.09; 2.67]	0.02
	very often	0.65 [0.29; 1.42]	0.28	1.02 [0.99; 1.04]	0.16	1.07 [0.99; 1.14]	0.08	1.78 [0.86; 3.69]	0.12
Sugary beverages	never	–	–	–	–	–	–	–	–
	rarely	0.75 [0.51; 1.11]	0.15	0.99 [0.97; 0.99]	0.03	1.01 [1.02; 1.09]	0.01	1.20 [0.88; 1.65]	0.25
	sometimes	0.59 [0.38; 0.92]	0.02	0.96 [0.94; 0.97]	< 0.001	1.09 [1.04; 1.14]	< 0.001	1.48 [1.01; 2.15]	0.04
	often	0.50 [0.28; 0.91]	0.02	0.94 [0.91; 0.96]	< 0.001	1.12 [1.05; 1.18]	< 0.001	2.05 [1.21; 3.46]	0.007
	very often	0.44 [0.22; 0.88]	0.02	0.94 [0.90; 0.97]	< 0.001	1.15 [1.08; 1.22]	< 0.001	2.54 [1.31; 4.92]	0.006
Energy drinks	never	–	–	–	–	–	–	–	–
	rarely	0.56 [0.31; 1.03]	0.06	0.98 [0.96; 1.00]	0.12	1.06 [0.99; 1.16]	0.06	1.07 [0.61; 1.87]	0.82
	sometimes	1.03 [0.38; 2.84]	0.95	0.93 [0.89; 0.98]	0.0037	1.11 [1.04; 1.19]	0.0036	2.67 [1.13; 6.29]	0.02
	often	0.51 [0.19; 1.41]	0.19	0.91 [0.85; 0.97]	0.007	1.10 [1.01; 1.21]	0.04	2.97 [1.01; 1.21]	0.03
	very often	0.57 [0.21; 1.54]	0.26	0.93 [0.88; 0.99]	0.02	1.05 [0.95; 1.16]	0.34	2.39 [0.95; 5.97]	0.06
Coffee	never	–	–	–	–	–	–	–	–
	rarely	1.25 [0.70; 2.22]	0.46	1.03 [1.01; 1.05]	0.006	1.04 [0.98; 1.10]	0.18	1.03 [0.65; 1.64]	0.89
	sometimes	0.90 [0.57; 1.42]	0.64	1.05 [1.03; 1.07]	< 0.001	1.02 [0.97; 1.08]	0.35	0.82 [0.55; 1.22]	0.32
	often	1.21 [0.80; 1.83]	0.38	1.04 [1.03; 1.06]	< 0.001	1.01 [0.97; 1.05]	0.70	1.25 [0.89; 1.73]	0.19
	very often	1.19 [0.78; 1.81]	0.42	1.03 [1.01; 1.04]	< 0.001	1.06 [1.02; 1.11]	0.005	2.16 [1.55; 3.02]	< 0.001

^a^Reference = “men”

^*b*^*OR *odds ratio*, CI *confidence interval

^c^Multinomial logistic regression analysis adjusted for age, BMI, and being a self-identified stress-eater; *p-value *<  0.004 is considered as statistically significant

^d^Multinomial logistic regression analysis adjusted for sex, BMI, and being a self-identified stress-eater; *p-value < * 0.004 is considered as statistically significant

^e^Multinomial logistic regression analysis adjusted for sex, age, and being a self-identified stress-eater;* p-value <  *0.004 is considered as statistically significant

^f^Reference = “self-identified non-stress-eaters”

^g^Multinomial logistic regression analysis adjusted for sex, age, and BMI; p-value <  0.004 is considered as statistically significant

### Substitutes

Table 5 shows which substitutes were considered as alternatives for the comfort foods “chocolate”, “cookies”, and “coffee”, that were stated to be consumed often and very often by at least 20% of the total survey population. The most frequently considered alternative food for chocolate was fresh fruits (74.4%) followed by dark chocolate (69.8%). For the comfort food “cookies”, the participants’ most favourite substitutes were fresh fruits (62.6%) and nuts-fruits-mixtures (55.2%). Tea without added sugar (64.4%) followed by water (47.0%) were the two most often stated substitutes for coffee.

**Table 5:** Consideration of pre-selected food as substitutes for chocolate, cookies, and coffee (*N* = 1096)^a^

	Total*	Females	Males	Stress-eaters
Substitute for …	n (%)	n (%)	n (%)	n (%)
Chocolate				
Dried fruits, berries	585 (53.4)	484 (53.5)	101 (52.6)	308 (53.5)
Fresh fruits, berries	815 (74.4)	673 (74.4)	142 (74.0)	408 (70.8)
Dark chocolate	765 (69.8)	633 (70.0)	132 (68.8)	401 (69.6)
Chocolate milk without added sugar	215 (19.6)	179 (19.8)	36 (18.8)	133 (22.4)
Pudding, cream desserts	349 (31.8)	286 (31.6)	63 (32.8)	205 (35.6)
Yoghurt, curd without added sugar	522 (47.6)	425 (47.0)	97 (50.5)	265 (46.0)
Muesli/fruit bar	495 (45.2)	393 (43.5)	102 (53.1)	272 (47.2)
Fruit/energy balls	289 (26.4)	252 (27.9)	37 (19.3)	170 (29.5)
Chocolate fruits	518 (47.3)	458 (50.7)	60 (31.3)	296 (51.4)
Other	105 (9.6)	91 (10.1)	14 (7.3)	57 (9.9)
Cookies				
Dried fruits, berries	404 (44.8)	325 (43.4)	79 (51.6)	220 (43.4)
Crispbread, rice wafers, rusk	486 (53.9)	419 (55.9)	67 (43.8)	274 (54.0)
Muesli/fruit bar	459 (50.9)	376 (50.2)	83 (54.2)	272 (53.6)
Popcorn without salt or added sugar	207 (22.9)	179 (23.9)	28 (18.3)	132 (26.0)
Fresh fruits, berries	565 (62.6)	461 (61.5)	104 (68.0)	302 (59.6)
Nuts-fruits-mixtures	498 (55.2)	410 (54.7)	88 (57.5)	282 (55.6)
Other	18 (2.0)	15 (2.0)	3 (2.0)	10 (2.0)
Coffee				
Decaffeinated coffee	275 (32.7)	242 (35.5)	33 (20.9)	156 (36.4)
Tea without added sugar	541 (64.4)	450 (66.0)	91 (57.6)	263 (61.4)
Sparkling fruit juice	176 (21.0)	133 (19.5)	43 (27.2)	83 (19.4)
Infused water (with fruits, vegetables or herbs)	155 (18.5)	128 (18.8)	27 (17.1)	76 (17.8)
Chocolate milk without added sugar	102 (12.1)	77 (11.3)	25 (15.8)	54 (12.6)
Water	395 (47.0)	307 (45.0)	88 (55.7)	191 (44.6)
Light, zero drinks	145 (17.3)	112 (16.4)	33 (20.9)	95 (22.2)
Other	20 (2.4)	18 (2.6)	2 (1.3)	9 (2.1)

^*^Different sample sizes because substitutes could only be chosen if participants stated to consume the respective comfort food (“rarely” to “very often”)

^a^Could you imagine eating the following food instead of [comfort food] in stressful situations?, Multiple choices were allowed

The odds of considering chocolate fruits as substitute for chocolate (OR = 2.07, *p* <  0.001) was two times higher for women than for men. Age was associated with four different substitutes for chocolate (Table 6). No association was found between BMI and considering substitutes for chocolate, cookies, and coffee (*p* > 0.002). Self-identified stress-eaters stated more often to consider light/zero drinks instead of coffee (OR = 2.11, *p* <  0.001) compared to non-stress-eaters (Table 6).

**Table 6:** Substitutes for chocolate, cookies, and coffee according to sex, age, BMI, and being a stress-eater

		Sex^b^		Age		BMI		Stress-eaters^g^	
Comfort food	Substitute^a^	OR [95% CI]^c^	p^d^	OR [95% CI]	p^e^	OR [95% CI]	p^f^	OR [95% CI]	p^h^
Chocolate	Dried fruits, berries	0.99 [0.71; 1.36]	0.93	1.00 [0.99; 1.01]	0.97	0.98 [0.95; 1.01]	0.13	1.06 [0.82; 1.36]	0.66
	Fresh fruits, berries	1.06 [0.73; 1.53]	0.76	0.98 [0.97; 0.99]	0.003	0.98 [0.95; 1.02]	0.35	0.68 [0.51; 0.91]	0.009
	Dark chocolate	1.01 [0.71; 1.43]	0.95	0.99 [0.98; 1.00]	0.11	0.98 [0.95; 1.01]	0.24	1.01 [0.77; 1.33]	0.92
	Chocolate milk without added sugar	0.97 [0.65; 1.48]	0.88	0.98 [0.97; 0.99]	0.003	1.02 [0.98; 1.06]	0.35	1.55 [1.12; 2.14]	0.008
	Pudding, cream desserts	0.88 [0.63; 1.25]	0.47	0.99 [0.98; 1.00]	0.07	1.01 [0.98; 1.05]	0.37	1.41 [1.08; 1.86]	0.01
	Yoghurt, curd without added sugar	0.88 [0.64; 1.22]	0.44	1.00 [0.99; 1.01]	0.95	0.99 [0.96; 1.02]	0.66	0.90 [0.70; 1.15]	0.40
	Muesli/fruit bar	0.61 [0.43; 0.84]	0.003	0.98 [0.97; 0.99]	< 0.001	0.99 [0.96; 1.02]	0.58	1.28 [0.99; 1.66]	0.05
	Fruit/energy balls	1.45 [0.98; 2.19]	0.07	0.98 [0.96; 0.99]	< 0.001	0.99 [0.95; 1.02]	0.55	1.37 [1.03; 1.83]	0.03
	Chocolate fruits	2.07 [1.47; 2.94]	< 0.001	0.97 [0.96; 0.98]	< 0.001	0.99 [0.96; 1.02]	0.60	1.32 [1.02; 1.70]	0.04
	Other	1.46 [0.81; 2.79]	0.23	1.04 [1.03; 1.06]	< 0.001	0.97 [0.92; 1.02]	0.29	1.15 [0.74; 1.77]	0.54
Cookies	Dried fruits, berries	0.68 [0.47; 0.98]	0.04	1.00 [0.99; 1.02]	0.36	0.96 [0.93; 1.00]	0.04	1.00 [0.76; 1.33]	0.98
	Crispbread, rice wafers, rusk	1.67 [1.16; 2.41]	0.006	0.99 [0.98; 1.00]	0.01	1.00 [0.97; 1.04]	0.87	0.91 [0.67; 1.21]	0.54
	Muesli/fruit bar	0.74 [0.51; 1.06]	0.10	0.99 [0.98; 1.00]	0.01	0.97 [0.94; 1.01]	0.13	1.40 [1.05; 1.86]	0.02
	Popcorn without salt or added sugar	1.27 [0.81; 2.04]	0.31	0.98 [0.97; 1.00]	0.02	1.00 [0.96; 1.04]	0.91	1.42 [1.01; 2.00]	0.04
	Fresh fruits, berries	0.78 [0.53; 1.14]	0.21	0.99 [0.98; 1.00]	0.09	0.98 [0.95; 1.02]	0.34	0.78 [0.58; 1.05]	0.10
	Nuts-fruits-mixtures	0.83 [0.58; 1.20]	0.33	1.01 [0.99; 1.02]	0.14	0.97 [0.94; 1.00]	0.10	1.14 [0.86; 1.51]	0.37
	Other	1.02 [0.31; 4.62]	0.98	1.03 [1.00; 1.07]	0.04	0.98 [0.85; 1.08]	0.69	1.09 [0.40; 3.07]	0.87
Coffee	Decaffeinated coffee	1.90 [1.25; 2.96]	0.003	1.01 [1.00; 1.02]	0.03	0.98 [0.95; 1.02]	0.27	1.37 [1.01; 1.87]	0.05
	Tea without added sugar	1.41 [0.97; 2.04]	0.07	1.00 [0.98; 1.01]	0.39	0.95 [0.92; 0.99]	0.007	0.78 [0.58; 1.06]	0.11
	Sparkling fruit juice	0.74 [0.49; 1.13]	0.15	0.99 [0.98; 1.01]	0.40	1.05 [1.01; 1.09]	0.01	0.78 [0.55; 1.12]	0.18
	Infused water	1.15 [0.72; 1.88]	0.56	1.00 [0.99; 1.01]	1.00	1.00 [0.96; 1.04]	0.94	0.89 [0.62; 1.29]	0.55
	Chocolate milk without added sugar	0.67 [0.41; 1.14]	0.13	0.98 [0.97; 1.00]	0.07	1.03 [0.98; 1.07]	0.30	1.10 [0.71; 1.72]	0.68
	Water	0.68 [0.47; 0.97]	0.03	1.00 [0.99; 1.02]	0.43	1.00 [0.97; 1.03]	0.97	0.88 [0.67; 1.18]	0.39
	Light, zero drinks	0.62 [0.39; 0.99]	0.04	0.99 [0.97; 1.00]	0.11	1.03 [0.99; 1.07]	0.14	2.11 [1.42; 3.16]	< 0.001
	Other	2.37 [0.64; 15.32]	0.26	1.01 [0.97; 1.04]	0.74	1.01 [0.90; 1.11]	0.86	0.70 [0.27; 1.79]	0.45

^a^Reference = “not considered”

^b^Reference = “men”

^*c*^*OR *odds ratio*, CI *confidence interval

^d^Binary logistic regression analysis adjusted for age, BMI, and being a self-identified stress-eater;* p-value *< 0.002 is considered as statistically significant

^e^Binary logistic regression analysis adjusted for sex, BMI, and being a self-identified stress-eater;* p-value *< 0.002 is considered as statistically significant

^f^Binary logistic regression analysis adjusted for sex, age, and being a self-identified stress-eater;* p-value *< 0.002 is considered as statistically significant

^g^Reference = “self-identified non-stress-eaters”

^f^Binary logistic regression analysis adjusted for sex, age, and BMI; p-value < 0.002 is considered as statistically significant

## Discussion

This survey shows that more than half of the survey participants change often or very often their eating behaviour in response to stress. The two most favourite comfort foods were chocolate and coffee. The consideration of substitutes for chocolate, cookies, and coffee was heterogeneous with different substitutes per comfort food.

According to the literature, about 50% of stress-eaters eat more and about 50% of stress-eaters eat less than normal (e.g. [4]). In contrast, 68.9% of the self-reported stress-eaters in this survey population stated to be stress-overeaters.

Chocolate and coffee were by far the two most favoured comfort foods, followed by cookies. This finding confirms the result of a study conducted in the USA that identified chocolate as the most frequently mentioned sweet comfort food [22]. A previous survey completed by students in the USA has reported a significantly positive association of perceived stress with coffee consumption [16]. The fact that only two out of 13 comfort foods were stated to be consumed often or very often by our survey participants could indicate that stress-eating behaviour does not vary much from person to person. Furthermore, experiencing stress as well as eating behaviour per se is very subjective and it might be difficult for participants to figure out that dietary patterns and food consumption change in response to stress.

The offered substitutes per comfort food were diverse. Some substitutes were very similar to the respective comfort food (e.g. dark chocolate instead of chocolate). Other substitutes were very different from the comfort food (e.g. fresh fruits instead of cookies). The fact that all offered substitutes for the three most favourite comfort foods could be imagined more or less by the survey participants as healthier alternative might indicate that these substitutes may be suitable for practical terms in real life. However, this assumption needs to be examined in an intervention study. We further found that rather healthy substitutes were stated more often compared to others (e.g. fresh fruits instead of chocolate and cookies, tea without added sugar instead of coffee). This fact could be used to address the issue of “stress-eating” comprehensively with nudging, an approach to change food choices. A systematic review showed its ability to promote healthy food choices by changing the order of food products or their proximity [27]. However, it cannot be ruled out that our survey participants made these healthy substitute choices due to social desirability, as participants were only asked if they could imagine to eat the offered substitutes.

Regarding the role of sex for the reported consumption frequency of comfort foods, we found that chocolate was more frequently consumed by women in stressful situations than by men, which is in line with previous findings [5, 15, 18]. Looking at substitutes, women stated more often to consider chocolate fruits instead of chocolate than men.

Age was partially associated with comfort food consumption. For example older people stated more often to consume frequently coffee during stress. Furthermore, older people stated less often to accept selected substitutes for chocolate, but more often to consider self-named substitutes than younger people. This could be a hint that older people have different preferences regarding substitutes.

A higher BMI was associated with a frequent consumption of fried food, hamburgers, and sugar-sweetend beverages. This result is plausible since individuals with overweight or obesity often experience emotional stress like social exclusion and shame [28], which could promote emotional eating. Previous studies examing the relationship between perceived stress, eating behaviour, and obesity differ in their study design (e.g. cross-sectional, longitudinal), methods, and research question. In a cross-sectional study by Richardson et al. including 101 American women with children, perceived stress was positively associated with uncontrolled and emotional eating, and stress with severe obesity, but independently from eating behaviour and quality [29]. A longitudinal, population-based study with almost 6000 participants in Finland could show for women at the age of 31 years that stress-eaters had the highest BMI and that stress-eating was associated with obesity [30]. In general, more longitudinal studies are needed to be able to assess the causal relationship between stress-eating and obesity.

Because of these findings the issue of “stress-eating” should be tackled to protect people at risk. It has to be mentioned that the present survey has not collected data about the quantity of comfort food consumption and the causality for obesity is, therefore, speculative. The focus of this survey was on collecting data about comfort foods per se and on examing whether and what healthier substitutes the survey participants could imagine to choose.

The findings of this survey on stress-eating behaviour provide new insights into potential new strategies to address this frequent cause of high-caloric and unhealthy food intake. However, there is need to perform prospective and intervention studies to explore how stress-related consumption of popular comfort foods can be replaced by healthier food alternatives, and how approaches like nudging and personalised dietary recommendations can be best employed to achieve the consumption of healthy food during stress.

This online survey is the first examining the topic of “comfort foods” and “suitable healthy substitutes” among adults in Germany. The data has been collected with a standardised questionnaire developed by an interdisciplinary team. Although the sample size is rather large, the validity is limited by the non-representative study population consisting of a majority of females and students, and by the fact that all data are self-reported. In addition, a set of pre-selected comfort foods was presented to the participants and they could not specify any others. In addition, the consideration of specific foods as healthier substitutes was theoretical, which allows no conclusion whether these substitutes would work in real life. Therefore, the knowledge about comfort foods and substitutes should be explored in intervention studies to examine whether stress-eaters consume substitutes and whether eating behaviour in stressful situations can be improved. Lastly, it is noteworthy that the data collection was performed during the Covid-19 pandemic, which is associated with changed food consumption [31]. This survey did not focus on stress-eating during the pandemic period or collected detailed data on the reason of perceived stress but aimed to collect data about stress and stress-induced eating in general.

## Conclusions

According to this survey performed during the Covid-19 pandemic, specific comfort foods and substitutes are preferred in stressful situations. The consumption frequency of comfort foods and the selection of substitutes seem to be associated with sex, age, BMI, and being a self-identified stress-eater. The findings should be confirmed in further studies, especially in intervention studies.

## Supplementary Information

**Additional file 1: Supplementary results**. **Suppl. Table 1.** Stress-eating behaviour of women and men who identified themselfes as stress-eaters. **Suppl. Table 2.** Sub-group specific consumption frequency of comfort foods in stressful situations.

## Data Availability

The datasets used and/or analyzed during this survey are available from the corresponding author upon reasonable request.

## Abbreviations

BMI: Body mass index
CI: Confidence interval
OR: Odds ratio
