# Supplementary material for: Stress eating: an online survey of eating behaviours, comfort foods, and healthy food substitutes in German adults
Source: BMC Public Health. 2022 Feb 24;22:391. doi: 10.1186/s12889-022-12787-9 (PMC8868035; doi:10.1186/s12889-022-12787-9)
Supplement: Supplementary file 1 — Additional file 1: Supplementary results. Suppl. Table 1. Stress-eating behaviour of women and men who identified themselfes as stress-eaters. Suppl. Table 2. Sub-group specific consumption frequency of comfort foods in stressful situations. [file 12889_2022_12787_MOESM1_ESM.pdf]

Additional File 1: Supplementary results

Suppl. Table 1. Stress-eating behaviour of women and men who identified themselves as stress-eaters[a]

	**Never**	**Rarely**	**Sometimes**	**Often**	**Very often**
**"I eat …"**	n (%)	n (%)	n (%)	n (%)	n (%)
**Females (N = 527)**					
More* (N = 526)	14 (2.7)	25 (4.8)	117 (22.2)	243 (46.2)	127 (24.1)
Less* (N = 524)	175 (33.4)	191 (36.5)	93 (17.7)	44 (8.4)	21 (4.0)
More often	24 (4.6)	39 (7.4)	103 (19.5)	251 (47.6)	110 (20.9)
More rarely	206 (39.1)	183 (34.7)	73 (13.9)	48 (9.1)	17 (3.2)
Faster	60 (11.4)	72 (13.7)	107 (20.3)	169 (32.1)	119 (22.6)
Slower	304 (57.7)	147 (27.9)	54 (10.2)	17 (3.2)	5 (0.9)
Other food	44 (8.3)	60 (11.4)	131 (24.9)	171 (32.4)	121 (23.0)
**Males (N = 67)**					
More.* (N = 66)	2 (3.0)	7 (10.6)	19 (28.8)	27 (40.9)	11 (16.7)
Less.* (N = 66)	22 (33.3)	25 (37.9)	10 (15.2)	7 (10.6)	2 (3.0)
More often.	7 (10.4)	8 (11.9)	14 (20.9)	29 (43.3)	9 (13.4)
More rarely.	27 (40.3)	22 (32.8)	12 (17.9)	5 (7.5)	1 (1.5)
Faster.	3 (4.5)	7 (10.4)	15 (22.4)	23 (34.3)	19 (28.4)
Slower.	37 (55.2)	19 (28.4)	8 (11.9)	2 (3.0)	1 (1.5)
Other food.	6 (9.0)	7 (10.4)	23 (34.3)	18 (26.9)	13 (19.4)

**Different sample sizes because of uncompleted surveys.*

[a]What applies to you regarding stress-eating?

Suppl. Table 2. Sub-group specific consumption frequency of comfort foods in stressful situations [a]

	**Never**	**Rarely**	**Sometimes**	**Often**	**Very often**
	n (%)	n (%)	n (%)	n (%)	n (%)
**Females (N = 994)**					
Chocolate/confections	90 (9.1)	156 (15.7)	234 (23.5)	299 (31.7)	215 (21.6)
Candies/gummy bears	491 (49.4)	233 (23.4)	136 (13.7)	91 (9.2)	43 (4.3)
Ice cream	576 (58.0)	210 (21.1)	135 (13.6)	54 (5.4)	19 (1.9)
Cake	302 (30.4)	279 (28.1)	250 (25.2)	131 (13.2)	32 (3.2)
Cookies	245 (24.6)	218 (21.9)	277 (27.9)	192 (19.3)	62 (6.2)
Crisps/crackers	374 (37.6)	249 (25.1)	180 (18.1)	140 (14.1)	51 (5.1)
Salted nuts	496 (49.9)	195 (19.6)	172 (17.3)	99 (10.0)	32 (3.2)
Fried food/chips	623 (62.7)	192 (19.3)	102 (10.3)	62 (6.2)	15 (1.5)
Hamburgers etc.	541 (54.4)	215 (21.6)	137 (13.8)	80 (8.0)	21 (2.1)
Alcohol*	472 (47.6)	219 (22.1)	193 (19.5)	80 (8.1)	27 (2.7)
Sugary beverages	598 (60.2)	185 (18.6)	118 (11.9)	57 (5.7)	36 (3.6)
Energy drinks	892 (89.7)	42 (4.2)	26 (2.6)	16 (1.6)	18 (1.8)

Coffee	312 (31.4)	85 (8.6)	128 (12.9)	230 (23.1)	239 (24.0)
	**Never**	**Rarely**	**Sometimes**	**Often**	**Very often**
	n (%)	n (%)	n (%)	n (%)	n (%)
**Males (N = 240)**					
Chocolate/confections	48 (20.0)	65 (27.1)	45 (18.8)	57 (23.8)	25 (10.4)
Candies/gummy bears	136 (56.7)	50 (20.8)	31 (12.9)	17 (7.1)	6 (2.5)
Ice cream	156 (65.0)	54 (22.5)	24 (10.0)	5 (2.1)	1 (0.4)
Cake	111 (46.3)	53 (22.1)	56 (23.3)	16 (6.7)	4 (1.7)
Cookies	87 (36.3)	58 (24.2)	51 (21.3)	39 (16.3)	5 (2.1)
Crisps/crackers	100 (41.7)	54 (22.5)	44 (18.3)	32 (13.3)	10 (4.2)
Salted nuts	118 (49.2)	58 (24.2)	27 (11.3)	24 (10.0)	13 (5.4)
Fried food/chips	143 (59.6)	44 (18.3)	32 (13.3)	14 (5.8)	7 (2.9)
Hamburgers etc.	110 (45.8)	55 (22.9)	47 (19.6)	24 (10.0)	4 (1.7)
Alcohol	118 (49.2)	49 (20.4)	36 (15.0)	27 (11.3)	10 (4.2)
Sugary beverages	119 (49.6)	49 (20.4)	38 (15.8)	20 (8.3)	14 (5.8)
Energy drinks	205 (85.4)	18 (7.5)	5 (2.1)	6 (2.5)	6 (2.5)
Coffee	82 (34.2)	19 (7.9)	42 (17.5)	49 (20.4)	48 (20.0)
**Stress-eaters (N = 594)**					
Chocolate/confections	18 (3.0)	57 (9.6)	89 (15.0)	233 (39.2)	197 (33.2)
Candies/gummy bears	236 (39.7)	144 (24.2)	85 (14.3)	87 (14.6)	42 (7.1)
Ice cream	279 (47.0)	144 (24.2)	111 (18.7)	45 (7.6)	15 (2.5)
Cake	131 (22.1)	160 (26.9)	164 (27.6)	108 (18.2)	31 (5.2)
Cookies	87 (14.6)	108 (18.2)	170 (28.6)	168 (28.3)	61 (10.3)
Crisps/crackers	167 (28.1)	137 (23.1)	109 (18.4)	130 (21.9)	51 (8.6)
Salted nuts	250 (42.1)	124 (20.9)	108 (18.2)	74 (12.5)	38 (6.4)
Fried food/chips	322 (54.2)	124 (20.9)	73 (12.3)	58 (9.8)	17 (2.9)
Hamburgers etc.	273 (46.0)	118 (19.9)	110 (18.5)	72 (12.1)	21 (3.5)
Alcohol*	262 (44.2)	126 (21.2)	120 (20.2)	63 (10.6)	22 (3.7)
Sugary beverages	312 (52.5)	115 (19.4)	85 (14.3)	48 (8.1)	34 (5.7)
Energy drinks	510 (85.9)	29 (4.9)	23 (3.9)	16 (2.7)	16 (2.7)
Coffee	166 (27.9)	47 (7.9)	63 (10.6)	134 (22.6)	184 (31.0)

**Different sample size because of missing answers. Females: N = 991, Stress-eaters: N = 593*

[a]Think about the last month. How often did you eat the following food when you experienced stress?
